# Brainstem infarcts predict REM sleep behavior disorder in acute ischemic stroke

**DOI:** 10.1186/1471-2377-14-88

**Published:** 2014-04-23

**Authors:** Wai Kwong Tang, Dirk M Hermann, Yang Kun Chen, Hua Jun Liang, Xiang Xin Liu, Winnie Chui Wing Chu, Anil T Ahuja, Jill Abrigo, Vincent Mok, Gabor S Ungvari, Ka Sing Wong

**Affiliations:** 1 Department of Psychiatry, Chinese University of Hong Kong, Hong Kong, SAR, China; 2 Department of Neurology, Chair of Vascular Neurology, Dementia and Cognitive Health of the Elderly, University Hospital Essen, Essen, Germany; 3 Department of Neurology, Dongguan People’s Hospital, Dongguan, Guangdong, China; 4 Department of Imaging and Interventional Radiology, Chinese University of Hong Kong, Hong Kong, SAR, China; 5 Department of Medicine and Therapeutics, Chinese University of Hong Kong, Hong Kong, SAR, China; 6 University of Notre Dame Australia/Marian Centre, Perth, Australia; 7 School of Psychiatry and Clinical Neurosciences, University of Western Australia, Perth, Australia

**Keywords:** Sleep, Acute ischemic stroke, Ischemia, Brainstem, Infarcts

## Abstract

**Background:**

Rapid eye movement (REM) sleep behavior disorder (RBD) is a sleep disturbance in which patients enact their dreams while in REM sleep. The behavior is typically violent in association with violent dream content, so serious harm can be done to the patient or the bed partner. The prevalence of RBD is well-known in Parkinson’s disease, Lewy body dementia, and multiple systems atrophy. However, its prevalence and causes in stroke remained unclear. The aim of this study was to determine factors influencing the appearance of RBD in a prospective cohort of patients with acute ischemic stroke.

**Methods:**

A total of 2,024 patients with first-ever or recurrent acute ischemic stroke were admitted to the Acute Stroke Unit at the Prince of Wales Hospital between January 2010 and November 2011; 775 of them received an MRI scan. Within 2 days of admission, a research nurse collected demographic and clinical data and assessed the severity of each stroke using the National Institute of Health Stroke Scale (NIHSS). One hundred and nineteen of the 775 patients meeting study entry criteria formed the study sample. All eligible participants were invited to attend a research clinic 3 months after the onset of the index stroke. In the attendance, a research assistant administered the MMSE and the 13-item RBD questionnaire (RBDQ).

**Results:**

Among 119 stroke patients, 10.9% were exhibited RBD, defined as an REM sleep behavior disorder questionnaire score of 19 or above. The proportion of patients with acute brainstem infarct was significantly higher in RBD patients than those without RBD. Compared with patients without RBD, RBD patients were more likely to have brainstem infarcts and had smaller infarct volumes. In a multivariate analysis, in which stroke location and infarct volume were inserted, brainstem infarcts were an independent predictor of RBD (odds ratio = 3.686; *P* = 0.032).

**Conclusions:**

The results support the notion of a predominant role of brainstem injury in the development of RBD and suggest that patients with brainstem infarcts RBD should be evaluated by a clinical neurologist.

## Background

Sleep disturbances are frequently found in stroke [1-4]. They increase the risk of stroke [5,6] and affect the clinical course and outcome of stroke [1,7]. Functional impairment, longer hospitalization and rehabilitation periods have been reported in stroke patients with sleep disturbance [8]. Rapid eye movement (REM) sleep behavior disorder (RBD) is a sleep disturbance in which patients enact their dreams while in REM sleep. The behavior is typically violent in association with violent dream content, so serious harm can be done to the patient or the bed partner. RBD predominantly affects older adults and has an estimated prevalence of 0.4–0.5% in adults [9]. RBD may be idiopathic or part of a neurodegenerative condition, particularly Parkinson’s disease, Lewy body dementia, and multiple systems atrophy with a prevalence ranging from 13% to 100% [9,10]. In neurodegenerative diseases, RBD is associated with brainstem lesions [10].

The prevalence and pathophysiology of RBD in stroke are largely unknown. Only case reports and small case series have been published and suggest that RBD in stroke is related to brainstem lesions [11-15]. Specifically, pontine strokes were described in single case reports [11,13,15]. Three in a series of six patients with RBD had infarcts in the dorsal pontomesencephalon [12].

The aim of this study was to determine factors influencing the appearance of RBD in a prospective cohort of patients with acute ischemic stroke.

## Methods

### Participants

**Figure 1:**
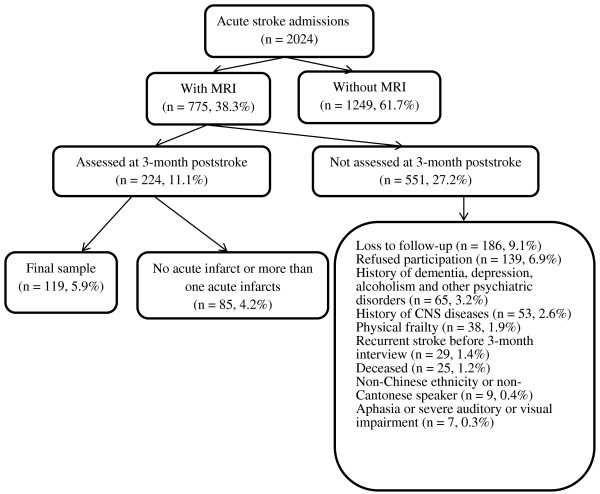
Recruitment profile of the study. *CNS* central nervous system, *MMSE* mini-mental state examination, *MRI* magnetic resonance imaging.

A total of 2,024 patients with first-ever or recurrent acute ischemic stroke were admitted to the Acute Stroke Unit at the Prince of Wales Hospital between January 2010 and November 2011; 775 of them received an MRI scan. All patients were screened for inclusion criteria (Figure 1): 1. Chinese ethnicity; 2. Cantonese as the primary language; 3. well-documented first or recurrent acute stroke occurring within 7 days before admission; and 4. the ability and willingness to give consent. The exclusion criteria were: 1. transient ischemic attack, cerebral hemorrhage, subdural hematoma or subarachnoid hemorrhage; 2. history of a CNS disease such as tumor, Parkinson’s disease, dementia, or others; 3. history of depression, alcoholism or other psychiatric disorders; 4. Mini-Mental State Examination (MMSE) [16] score of less than 20; 5. severe aphasia or auditory or visual impairment; 6. physical frailty; and 7. recurrence of stroke prior to the 3-month assessment; and 8. No acute infarct or more than one acute infarct in MRI.

### Materials and procedure

The study protocol was approved by the Clinical Research Ethics Committee of the Chinese University of Hong Kong. All eligible participants were invited to attend a research clinic 3 months after the onset of the index stroke, where they signed a consent form and received face-to-face interview conducted by a research assistant.

A research nurse collected demographic and clinical data, assessed the severity of each stroke using the National Institute of Health Stroke Scale (NIHSS) [17] within 2 days of admission, and entered these data in a Stroke Registry. The research assistant administered the MMSE 3 months after the onset of the index stroke. The research assistant, who was blind to the patients’ radiological data, also administered the 13-item RBD questionnaire (RBDQ) [18], which has a score ranging from 0 to 100.

The RBDQ demonstrated robust psychometric properties with good sensitivity (82.2%), specificity (86.9%), positive (86.3%) and negative (83.0%) predictive value, high internal consistency (90%), and test-retest reliability (89%) [18]. RBD was defined as the presence of clinically significant RBD symptoms indicated by an RBDQ score of 19 or above [18].

MRI was performed with a 1.5-T system within 7 days after admission. A neurologist (YKC), who was blind to the patients’ RBD status, assessed all of the MRIs. The number and size of acute infarcts affecting different structures, including the frontal, temporal, parietal and occipital lobes, subcortical white matter, thalamus, basal ganglia, brain stem and cerebellum were evaluated. If an infarct involved more than on location, e.g. basal ganglia and subcortical region, then it was counted twice, one for the basal ganglia and one for the subcortical white matter. The total area of acute infarcts on the DWI was measured by manual outlines of all areas with restricted water diffusion identified on the diffusion-weighted images with b values of 1000. The total volume was calculated by multiplying the total area by the sum of the slice thickness and gap [19]. The details of the MRI assessment have been described elsewhere [19].

### Statistical analysis

Patients without acute infarct or more than one acute infarct on MRI were excluded from the analysis. The demographic and clinical characteristics of the RBD patients (RBD group) were compared to those without RBD (non-RBD group). Subsequently, logistic regression models were constructed. In a multivariate regression, risk factors with a *P* value <0.05 were inserted using a forward stepwise selection strategy. Throughout the study, the significance threshold was set at *P* = 0.05.

## Results

One-hundred-nineteen of the 775 patients meeting the inclusion and exclusion criteria formed the study sample (Figure 1). There was no significant difference between included and excluded patients in terms of age, sex, and NIHSS score. Thirteen patients (10.9%) had RBD. In patients with brainstem infarct the prevalence of RBD was higher (6 out of 27; 22.2%). Four of the six (67%) of RBD patients with brainstem infarcts were men; five had ventral pontine infarct and one had medullar infarct.

The demographic and MRI characteristics and stroke-related data of the sample are shown in Table 1. The proportion of patients with acute brainstem and pontine base infarct was significantly higher in RBD patients than those without RBD. Compared to patients without RBD, RBD patients had smaller infarct volumes. The correlation between the presence of acute brainstem infarcts and acute pontine base infarcts was 0.901. The presence of acute brainstem infarct and infarct volume were entered into a multivariate logistic regression analysis where the presence of acute brainstem infarct was a significant independent predictor of RBD (odds ratio = 3.69; Table 2). Another regression model was constructed by entering the presence and volume of acute pontine infarcts. Acute pontine base infarct was not a significant predictor of RBD (Table 3).

**Table 1:** **Demographic characteristics**, **psychosocial risk factors**, **stroke severity**, **and radiological characteristics by RBD status**

	**RBD (n = 13) Mean ± SD**	**Non-RBD (n = 106) Mean ± SD**	***P***^**a**^
Age	67.3 ± 9.3	66.5 ± 10.7	0.796
Female sex	6 (46.2%)	41 (38.7%)	0.603^b^
Previous stroke	2 (15.4%)	7 (6.6%)	0.255^b^
NIHSS total score	4.9 ± 6.9	3.5 ± 3.1	0.624^c^
MMSE score	27.5 ± 2.7	27.5 ± 2.5	0.991^a^
Volume of acute infarct (ml)	0.7 ± 1.1	2.2 ± 5.1	0.020^c^
Presence of acute infarcts in			
Frontal	0 (0%)	12 (11.3%)	0.357^d^
Temporal	0 (0%)	2 (1.9%)	1.000^d^
Parietal	0 (0%)	3 (2.8%)	1.000^d^
Occipital	0 (0%)	1 (0.9%)	1.000^d^
Basal ganglia	1 (7.7%)	15 (14.2%)	1.000^d^
Thalamus	3 (23.1%)	14 (13.2%)	0.396^d^
Brainstem	6 (46.2%)	20 (19.8%)	0.036^b^
Midbrain	0 (0%)	1 (0.9%)	1.000^d^
Pons	5 (38.5%)	18 (17.0%)	0.128^d^
Pontine base	5 (38.4%)	17 (16.0%)	0.049^b^
Pontine tegmentum	0 (0.0%)	7 (6.6%)	1.000^d^
Coeruleus/subcoeruleus region	0 (0.0%)	1 (0.9%)	1.000^d^
Laterodorsal tegmental nuclei	0 (0.0%)	5 (4.7%)	0.946^d^
Medulla	1 (7.7%)	2 (1.9%)	0.295^d^
Cerebellum	0 (0%)	3 (2.8%)	1.000^d^
Subcortical white matter	3 (23.1%)	46 (43.4%)	0.160^d^

*MMSE* = Mini-Mental State Examination; *NIHSS* = National Institutes of Health Stroke Scale, *RBD* = *REM* sleep behavior disorder.

^a^t-test, ^b^chi-square test, ^c^Mann-Whitney U-test, ^d^Fisher’s exact test.

**Table 2:** Multivariate logistic model of the clinical determinants of RBD

**Variables**	**Odds ratio (95% C.I.)**	***P***^**a**^
Presence of brainstem infarct	3.686 (1.117 –12.164)	0.032
Volume of acute infarcts	-	0.389

RBD = REM sleep behavior disorder.

^a^Logistic regression.

**Table 3:** Multivariate logistic model of the clinical determinants of RBD

**Variables**	**Odds ratio (95% C.I.)**	***P***^**a**^
Presence of pontine base infarct	**-**	0.059
Volume of acute infarcts	**-**	0.358

RBD = REM sleep behavior disorder.

^a^Logistic regression.

## Discussion

This was the first systematic prospective examination of factors influencing the development of RBD in acute ischemic stroke. The main finding of the study is that brainstem infarcts are associated with RBD in acute ischemic stroke.

The frequency of RBD in this study was 10.9%, which is lower than found in other neurodegenerative diseases [9]. In patients with acute brainstem infarct, the frequency of RBD was 22% indicating that RBD may be common in this stroke subgroup. It is unknown whether RBD affects stroke outcome. Other parasomnias, such as restless leg syndrome, have been associated with increased mortality in the general population [20].

Stroke-related RBD is a secondary RBD [10]. There is evidence supporting the role of the brainstem in the pathogenesis of RBD in other neurological conditions [10,21]. Data from animal models suggest that RBD results from brainstem dysfunction leading to a lack of muscle atonia during REM sleep [9]. Within the brainstem, degeneration of the pontine glutamatergic and medullary GABAergic neurons has been implicated in the pathophysiology of RBD [22].

### Strengths and limitations of the study

The strength of this study is the prospective subject recruitment with MRI scans. Its main limitation is the small sample size, which was the consequence of stringent inclusion and exclusion criteria that allowed to obtain a well-defined sample. Thus, despite inclusion of only 119 patients, multivariate logistic regression could be performed, in which two factors, stroke topography and volume were included [23]. RBD was diagnosed using a questionnaire, and the questionnaire did not differentiate the onset of RBD before or after stroke. Ideally, polysomnography (PSG) should have been performed, but for logistical reasons PSG could not be included in this study.

## Conclusions

In conclusion, results of this study indicate that brainstem infarcts are associated with RBD in acute ischemic stroke. RBD should be evaluated by a clinical neurologist in patients with brainstem infarcts, given that RBD can easily be diagnosed and treated with clonazepam [24].

## Competing interests

The authors report no conflict of interest. The funding agencies had no role in study design, data collection and analysis, decision to publish or preparation of the manuscript.

## Authors’ contributions

WKT designed the study. YKC, HJL, XXL, WCWC, ATA, JA, VCTM, KSW conducted data collection. YKC, HJL conducted statistical analysis. WKT, HJL, XXL interpreted the data. WKT wrote the first draft of the manuscript. The critical revision of the manuscript was made by DMH, GSU. All authors reviewed the first draft of the paper. All authors read and approved the final manuscript.

## Pre-publication history

The pre-publication history for this paper can be accessed here:

http://www.biomedcentral.com/1471-2377/14/88/prepub
